# Outpatient weekly neoadjuvant chemotherapy followed by radiotherapy for advanced nasopharyngeal carcinoma: high complete response and low toxicity rates

**DOI:** 10.1038/sj.bjc.6600716

**Published:** 2003-01-28

**Authors:** J-C Lin, J-S Jan, C-Y Hsu, R-S Jiang, W-Y Wang

**Affiliations:** 1 Department of Radiation Oncology, Taichung Veterans General Hospital, Taichung, Taiwan; Institute of Clinical Medicine, College of Medicine, National Yang-Ming University, Taipei, Taiwan; Department of Otorhinolaryngology, Taichung Veterans General Hospital, Taichung, Taiwan; Department of Basic Medicine, Hung Kuang Institute of Technology, Taichung, Taiwan

**Keywords:** nasopharyngeal carcinoma, neoadjuvant, chemotherapy, radiotherapy

## Abstract

Nasopharyngeal carcinoma (NPC) is a radiosensitive and chemosensitive tumour. The aim of this prospective study is to evaluate the toxicity and efficacy of an outpatient weekly neoadjuvant chemotherapy (NeoCT) plus radiotherapy for advanced NPC. From November 1998 to August 2001, 90 NPC patients meeting the following criteria were treated: (1) neck node >6 cm; (2) supraclavicular node metastasis; (3) skull base destruction/intracranial invasion plus multiple nodes metastasis; (4) multiple neck nodes metastasis with one of nodal size >4 cm; or (5) elevated serum LDH level. The NeoCT consists of cisplatin 60 mg m^−2^, alternating with 5-fluorouracil 2500 mg m^−2^ plus leucovorin 250 mg m^−2^ (P–FL) by an outpatient weekly schedule for a total of 10 weeks. Local radiotherapy ⩾70 Gy by conventional fractionation was delivered within 1 week after NeoCT. Patient compliance was rather good. Grade 3–4 toxicity of NeoCT included leucopaenia (7.8%), anaemia (18.9%), thrombocytopaenia (3.3%), nausea/vomiting (4.4%), and weight loss (1.1%). Response evaluated after NeoCT showed 73.3% complete response (CR) rate of primary tumour, 71.1% CR rate of neck nodes, and an overall CR rate of 57.8%. In all, 88 out of 90 patients received rebiopsy of primary tumour and 55 patients (62.5%) revealed pathological CR. After a median follow-up time of 24 months, one persistent disease and 18 relapses were noted. The 2-year nasopharynx disease-free, neck disease-free, distant disease-free, overall, and progression-free survival rates are 98.9, 95.9, 80.0, 92.1, and 77.5%, respectively. Preliminary data of the current study show that P–FL NeoCT plus radiotherapy is a low-toxic regimen with promising results on very advanced NPC patients and merits to be investigated in phase III trials.

Nasopharyngeal carcinoma (NPC) has several characteristics that distinguish it from other head and neck malignancies. It is a geographically endemic, Epstein–Barr virus-associated carcinoma of epidermoid origin. The NPC cells are poorly differentiated or undifferentiated with a high incidence of lymphatic and haematologic dissemination, and have greater radiosensitivity. Radiotherapy is the most effective treatment for NPC because anatomic constraints make surgery difficult. Although early-stage NPC is highly radiocurable, the treatment results of locoregionally advanced NPC have been disappointing.

Combined chemoradiotherapy has been accepted by most oncologists as the standard treatment of advanced NPC. There is still great controversy, however, regarding the optimal drugs, timing, dosage, and duration of chemotherapy. In general, there are three different ways to incorporate chemotherapy into a curative course of radiotherapy: before (neoadjuvant), during (concurrent), and after (adjuvant) radiation therapy. Each mode of combined therapy has advantages and disadvantages, and has been extensively investigated during past years. The major flaws of neoadjuvant chemotherapy (NeoCT) are triggering of accelerated repopulation and crossresistance during subsequent radiotherapy. The dose intensity of concurrent chemotherapy that can be delivered safely during 7–8 weeks radiotherapy is usually lower than chemotherapy alone. This may compromise its efficacy in eradication of micrometastasis. Poor compliance and compromised blood supply are the two major problems of adjuvant chemotherapy. In this prospective study, we designed a novel schedule of outpatient weekly NeoCT plus radiotherapy for very advanced NPC.

## Patients and methods

In our hospital, all patients with biopsy-proven NPC are subjected to careful staging and evaluation before treatment. This included clinical examination of the head and neck region, fibre nasopharyngoscopy, computed tomography (CT) scan or magnetic resonance imaging (MRI) from the skull base to the whole neck, chest radiography, whole-body bone scan, abdominal sonography, complete blood count with differential count, platelet count, biochemical profile, and EBV serology. Chest CT scan and bone marrow biopsy were not routine, but selectively performed when suspicion of lung metastasis in chest radiography and abnormal blood routine were noted. Cancer stage was defined according to the American Joint Committee on Cancer (AJCC) 1997 staging system.

### Patient selection for NeoCT

According to our past experience, patients with one of the following criteria were found to have a high incidence of distant failure: (1) neck nodal size >6 cm; (2) supraclavicular node metastasis; (3) skull base destruction/intracranial invasion plus multiple nodes metastasis; (4) multiple neck nodes metastasis with one of nodal size >4 cm, and (5) elevated serum LDH level. From November 1998 to August 2001, 90 NPC patients meeting our selection criteria received NeoCT plus radiotherapy after obtaining informed consent. Other eligibility criteria were: (1) Karnofsky performance status ⩾60%; (2) white blood cell (WBC) count >3000 *μ*l^−1^ and platelet count >100 000 *μ*l^−1^; (3) serum creatinine level <2.0 mg dl^−1^; (4) normal liver function with total bilirubin <2.5 mg dl^−1^; and (5) no detectable distant metastasis.

### Treatment schedule

All patients received a subcutaneous implanted port. Weekly P–FL NeoCT consisted of cisplatin 60 mg m^−2^ 2-h infusion after hydration and antiemetics, alternating with 5-fluorouracil (5-FU) 2500 mg m^−2^ plus leucovorin 250 mg m^−2^, mixed in 240 ml of normal saline by continuous intravenous infusion for 24 h using an ambulatory pump in an outpatient setting. The NeoCT will be delayed if Grade 4 toxicity developed, and resumed after recovery. No dose modification was made and 10 weekly doses were planned. There were no problems with the subcutaneous port for chemotherapy administration except for the occurrence of catheter obstruction in one patient who needed surgical revision.

Radiotherapy was started within 1 week after completion of NeoCT using a linear accelerator of 6 MV photons by the same technique and fractionation as described previously (Lin and Jan, 1999), except for the incorporation of a 3-D conformal beam for the last 10–14 Gy. The total dose to the primary tumour is 70 Gy for T1-3 and 74 Gy for T4 disease. During the initial period of this trial (before June 1999), two courses of concurrent chemotherapy comprising 96-h continuous infusion of cisplatin 15 mg m^−2^ day^−1^ +5-FU 300 mg m^−2^ day^−1^ were planned at the first and fifth weeks of radiotherapy. Another 10-weekly postradiation adjuvant chemotherapy (cisplatin 25 mg m^−2^+5-FU 1250 mg m^−2^+bleomycin 10 mg m^−2^+leucovorin 120 mg m^−2^) was also recommended 2 months after radiotherapy. We discontinued the concurrent and adjuvant chemotherapy since June 1999 because of poor patient compliance.

### Patient assessments

Chemotherapy toxicity and tumour response were assessed according to the World Health Organization (WHO) criteria (Miller *et al*, 1981). Complete response (CR) was defined as the complete disappearance of all clinical and radiographic evidence of disease at the time of objective reevaluation. Partial response (PR) was defined as a ⩾50% decrease in the sum of the products of the greatest dimensions of all measurable lesions. Included in the definition of PR were no new lesions and no progression of any existing lesions. Stable disease (SD) was defined as a reduction in tumour size less than PR and an increase in tumour size less than that defined as progressive disease (PD) or no response. PD was defined as ⩾25% increase in total tumour size of ⩾1 lesion, or the appearance of a new lesion.

All patients were routinely assessed by indirect mirror examination for nasopharynx, palpation of neck nodes, measurement of body weight, complete blood count and platelet count once a week, or when patients experienced suffering and requested check-up during the course of chemoradiotherapy. CT scan of nasopharynx and neck was usually repeated at the ninth to tenth week for evaluation of tumour response and radiotherapy planning. Rebiopsy of nasopharynx under fibroscopy was performed before radiotherapy. Liver and renal function tests were rechecked before and after radiotherapy. After completion of the whole treatment, patients were followed every 2 months during the first year, every 3 months for the second and third years, and every 6 months thereafter. CT scan, chest radiograph, abdominal sonography, whole-body bone scan, blood count, and biochemistry tests were routinely performed annually or at the time of clinical suggestion of tumour relapse.

## Results

### Patient characteristics

Table 1

**Table 1:** Patient characteristics

**Characteristics**		**No. of cases**	**%**
Age (years)			
Range	24–71		
Median	43		
Mean	45		
			
Sex			
Male		66	73.3
Female		24	26.7
			
Karnofsky scale			
⩾80%		82	91.1
<80%		8	8.9
			
Pathology (WHO classification)			
Type I		4	4.4
Type II		70	77.8
Type III		16	17.8
			
T-stage (1997 AJCC)			
T1		6	6.7
T2a		2	2.2
T2b		19	21.1
T3		12	13.3
T4		51	56.7
			
N-stage (1997 AJCC)			
N2		56	62.2
N3		34	37.8
			
Concurrent chemotherapy			
Yes		11	12.2
No		79	87.8
			
Adjuvant chemotherapy			
Yes		11	12.2
No		79	87.8

lists the pretreatment patient and tumour characteristics for the 90 patients. Although one of our eligibility criteria was Karnofsky performance status of 60 or greater, most of our patients (91.1%) belong to a performance status of 80–100. The TNM stage distribution of our patients (1997 AJCC T1/T2a/T2b/T3/T4=6/2/19/12/51 and N2/N3=56/34) demonstrates very advanced stage disease.

### Patient compliance to P–FL NeoCT

Of 90 patients, 84 finished the planned 10-week P–FL NeoCT without interruption. Two patients stopped NeoCT prematurely after seven doses because of their refusal, with complete regression of their tumour clinically and pathologically. One patient received two more doses (12 doses) than planned because of a 4-week interruption by earthquake and personal affair. Another patient escaped after 8-week NeoCT with clinical complete remission. The tumour relapsed 11 months later and he received 10-week NeoCT followed by radiotherapy as schedule. Except for four treatment interruptions during NeoCT mentioned above, two additional patients completed the planned NeoCT with a 1-week delay because of gastrointestinal bleeding and operation for suspected intra-abdominal metastasis, but proven as benign tumour arising from the lesser omentum.

### Toxicity of P–FL NeoCT

Acute toxicity was mild and well tolerated (Table 2

**Table 2:** Acute toxicities of neoadjuvant P–FL chemotherapy

	**Grade**				
**Toxicity**	**0**	**1**	**2**	**3**	**4**
Leucopaenia	21 (23.3%)	30 (33.3%)	32 (35.6%)	7 (7.8%)	0
Anaemia	15 (16.7%)	34 (38.9%)	24 (26.7%)	14 (15.6%)	3 (3.3%)
Thrombocytopaenia	82 (91.1%)	2 (2.2%)	4 (4.4%)	2 (2.2%)	1 (1.1%)
Mucositis	87 (96.7%)	1 (1.1%)	2 (2.2%)	0	0
Nausea/vomiting	40 (44.4%)	33 (36.7%)	13 (14.4%)	4 (4.4%)	0
Weight loss	42 (46.7%)	33 (36.7%)	14 (15.6%)	1 (1.1%)	0
Alopecia	86 (95.6%)	4 (4.4%)	0	0	0

). Some patients experienced a mild degree of anorexia and weakness during the NeoCT. Nausea and emesis occurred in 50 of the 90 patients during their treatment, but was usually mild (only four patients experienced Grade 3 vomiting). Mucositis was observed in only three patients of Grade 1–2. In all, 96% of the patients had no observable hair loss and only 4% patients experienced Grade 1 alopecia. No patients complained of diarrhoea. Body weight loss was defined as the difference between prechemotherapy body weight and the nadir body weight during chemotherapy. About half of the patients experienced body weight loss of mild degree (only 1.1% belong to Grade 3). There was no liver or renal function impairment. Haematological toxicity was also mild. Grades 3–4 toxicities occurred in 7.8% of patients for leucopaenia, 3.3% of patients for thrombocytopaenia, and 18.9% of patients for anaemia.

### Tumour response to P–FL NeoCT

At the end of NeoCT, we observed 52 patients with CR (57.8%), 36 with PR (40.0%), and 2 with SD (2.2%), for an overall response rate of 97.8% (Table 3

**Table 3:** Tumour response

	**Site**		
**Response**	**Nasopharynx**	**Neck**	**Overall**
Clinical			
CR	66 (73.3%)	64 (71.1%)	52 (57.8%)
PR	23 (25.6%)	24 (26.7%)	36 (40.0%)
SD	1 (1.1%)	2 (2.2%)	2 (2.2%)
			
Pathological^a^			
CR	55 (62.5%)		
Residual tumour	33 (37.5%)		

a A total of 88 out of 90 patients received rebiopsy of nasopharynx after neoadjuvant chemotherapy before radiotherapy.

**Figure 1:**
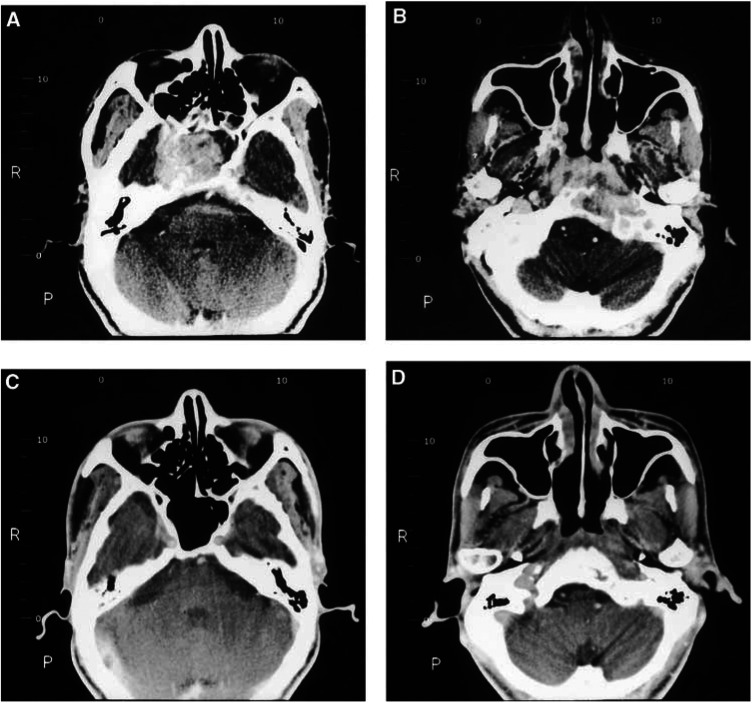
Pretreatment CT scan (**A, B**) showed a big nasopharyngeal tumour with intracranial invasion in a patient presenting as multiple cranial nerve palsy. The tumour regressed completely with bony regeneration of the destructed skull base after 10-week neoadjuvant chemotherapy (**C, D**).

CR=complete response, PR=partial response, SD=stable disease.

). Clinically, CR rates of primary tumour and neck nodes were 73.3% (66 out of 90) and 71.1% (64 out of 90), respectively. Figure 1

shows the complete disappearance of original huge NPC with intracranial invasion after 10-week P–FL NeoCT. A total of 88 out of 90 patients received rebiopsy before radiotherapy and pathologically CR was observed in 55 patients (62.5%).

### Toxicity and compliance to subsequent radiotherapy

All except one patient finished radiotherapy ⩾70 Gy. One patient presented with T4N3 tumour that responded poorly to NeoCT. He refused radiotherapy after 34 Gy with stable disease. Most patients received 70 (31 patients) and 74 Gy (54 cases). The average treatment duration of radiotherapy was 52 days (range 44–84 days). Only five patients completed local radiotherapy more than 9 weeks. Acute toxicity was moderate and manageable (Table 4

**Table 4:** Acute toxicities of subsequent radiotherapy

	**Grade**				
**Toxicity**	**0**	**1**	**2**	**3**	**4**
Leucopaenia	9 (10.0%)	26 (28.9%)	41 (45.6%)	14 (15.6%)	0
Anaemia	11 (12.2%)	32 (35.6%)	31 (34.4%)	9 (10.0%)	7 (7.8%)
Thrombocytopaenia	85 (94.4%)	0	3 (3.3%)	1 (1.1%)	1 (1.1%)
Mucositis	0	19 (21.1%)	24 (26.7%)	47 (52.2%)	0
Skin reaction	0	22 (24.4%)	44 (48.9%)	24 (26.7%)	0
Weight loss	22 (24.4%)	34 (37.8%)	33 (36.7%)	1 (1.1%)	0
Nausea/vomiting	84 (93.3%)	6 (6.7%)	0	0	0

). Since we have no gap between NeoCT and subsequent radiotherapy, chemotherapy-induced bone marrow suppression persisted during the period of the first to third weeks of radiotherapy. In all, 61% (55 out of 90) of patients encountered the nadir WBC count <3000 *μ*l^−1^, usually occurring in the first 3 weeks of radiotherapy. A total of 52% (47 out of 90) of patients experienced nadir haemoglobin <8 g%. The major toxicity induced by radiotherapy was mucositis with 52.2% Grade 3 and 26.7% Grade 2. Grades 3 and 2 skin reactions were noted in 26.7 and 48.9% of the patients, respectively.

### Compliance to additional concurrent/adjuvant chemotherapy

Before June 1999, additional concurrent/adjuvant chemotherapy was also planned after NeoCT. For the 17 patients intended to be treated during this period, the compliance to concurrent and/or adjuvant chemotherapy is poor. At the end of NeoCT, we did not recommend concurrent chemotherapy because of persistent leucopaenia <2500 *μ*l^−1^ in six out of 17 patients. CCRT was delivered for 11 out of 17 patients, but five patients needed more delay (>1 week) to start CCRT and four patients refused the second cycle of concurrent chemotherapy. Adjuvant chemotherapy was given for 11 out of 17 patients, 2 months after the completion of radiotherapy. Four of 11 patients had 1–4 weeks interruption during the adjuvant chemotherapy period and two patients refused further treatment after 6-week adjuvant chemotherapy. Thus, we modified our original three-phase chemotherapy to NeoCT+radiotherapy alone for the last 73 patients.

### Patterns of failure and survival

**Figure 2:**
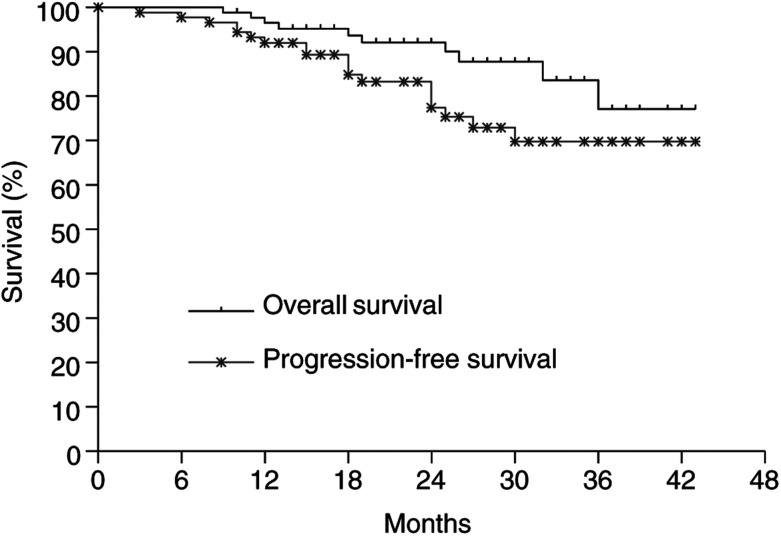
Overall and progression-free survival curves for all 90 patients.

The patient who received incomplete radiotherapy at 34 Gy was counted as locoregional failure. The current status of the remaining 89 patients, after a median follow-up of 24 (range 9–42) months, showed that two patients failed at primary, one patient failed regionally, one patient failed in neck and distant site, and 14 patients developed distant metastases alone. The 2-year nasopharynx disease-free, neck disease-free, and distant disease-free survival rates for all patients are 98.9, 95.9, and 80.0%, respectively. The 2-year overall and progression-free survival rates are 92.1 and 77.5%, respectively (Figure 2

).

**Figure 3:**
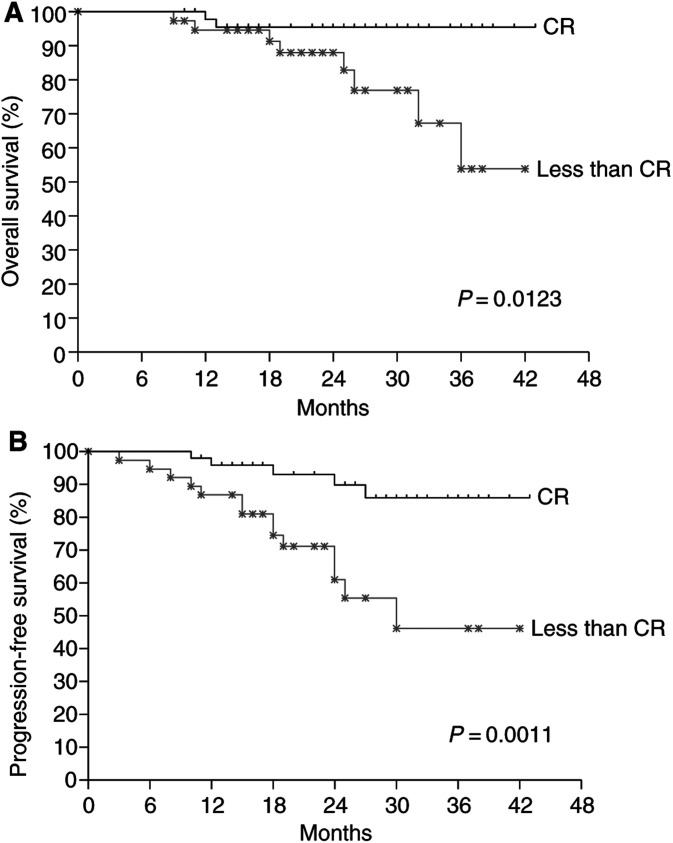
Comparison of overall survival (**A**) and progression-free survival (**B**) according to tumour response after neoadjuvant chemotherapy using the Kaplan–Meier estimate and the log-rank test. CR=complete response.

We also analysed the impact of additional concurrent/adjuvant chemotherapy on treatment outcome and found no statistically significant difference in terms of progression-free and overall survivals. When we evaluated the influence of tumour response after NeoCT, overall survival (Figure 3A

) and progression-free survival (Figure 3B) were significantly higher in the group who had a complete response than in the group who had less than a complete response.

### Late complications

The late complications that occurred up to the time of this writing were usually mild. All patients suffered from xerostomia of varying degrees. A total of 18 patients complained of hearing impairment. Six patients experienced neck fibrosis of different degrees. Six patients had trismus and four patients suffered from transient Lhermitte's sign. The severe late morbidities (⩾Grade 3–4) consist of temporal lobe oedema in one case, trismus 0.2 cm in one case, severe neck fibrosis in four patients, and marked hearing loss in six patients. Four of these six patients also received additional cisplatin-based chemotherapy as a salvage for distant metastases that might contribute to their hearing loss.

## Discussion

Treatment failures for advanced NPC in the past have been both high rates of local recurrence and distant metastasis. As a result of recent advances in radiation oncology and the combined use of chemotherapy, the patterns of failure have been predominantly because of distant metastasis (Huang *et al*, 1985; Lee *et al*, 1992; Chan *et al*, 1995; Cvitkovic and INCSG, 1996; Lin *et al*, 1996; Al-Sarraf *et al*, 1998; Chua *et al*, 1998; Cheng *et al*, 2000; Ma *et al*, 2001; Wolden *et al*, 2001). Adding more chemotherapy into original radiotherapy schedule for patients with high risk of distant failure is a reasonable and critical approach to enhance cure rate.

NeoCT for NPC had been tried more than 10 years ago, but seemed to be abandoned during recent years. Instead, concurrent chemoradiotherapy (CCRT) with adjuvant chemotherapy has become popular especially after the Intergroup report (Al-Sarraf *et al*, 1998). We agree that concurrent chemotherapy has a good timing and has some benefits, such as different cell-killing effect, avoidance of crossresistance, independent toxicity (if careful selection of the drug), and potentially additive or synergistic action between radiation and chemotherapy. However, it does not exclude the potential effect of a different chemotherapy schedule.

To the best of our knowledge, there have been nine phase III randomised trials to investigate the role of combined chemoradiotherapy in NPC so far (Rossi *et al*, 1988; Chan *et al*, 1995, 2002; Cvitkovic and INCSG, 1996; Al-Sarraf *et al*, 1998; Chua *et al*, 1998; Ma *et al*, 2001; Chi *et al*, 2002; Hareyama *et al*, 2002). Table 5

**Table 5:** Literature review of phase III studies in NPC

**Authors (published year)**	**Entry criteria**	**Treatment**	**No. of cases**	**Reported survival (%)**	**Estimated^a^ 2-year OS (%)**
Rossi *et al* (1988)	1978 UICC	RT	116	67 (4-year OS)	83
	Stage II–IV	RT+AdjCT	113	59	70
Chi *et al* (2002)	1988 AJCC/UICC	RT	77	60.5 (5-year OS)	80
	Stage IV	RT+AdjCT	77	54.5	78
Al-Sarraf *et al* (1998)	1988 AJCC/UICC	RT	69	37 (5-year OS*)	58
	Stage III–IV	CCRT+AdjCT	78	67	82
Chan *et al* (2002)	Ho's N2-3 or	RT	176	69 (2-year RFS)	
	node⩾4 cm	CCRT	174	76	
Chan *et al* (1995)	Ho's N3 or	RT	40	81 (2-year OS)	81
	node ⩾4 cm	NeoCT+RT+AdjCT	37	80	80
Cvitkovic and INCSG (1996)	1987 UICC	RT	168	54 (3-year OS)	60
	N2-3	NeoCT+RT	171	60	65
Chua *et al* (1998)	Ho's T3, N2-3	RT	167	71 (3-year OS)	81
	or node ⩾3 cm	NeoCT+RT	167	78	81
Ma *et al* (2001)	1992 Chinese	RT	228	56 (5-year OS)	82
	Stage III–IV	NeoCT+RT	228	63	87
Hareyama *et al* (2002)	1988 AJCC/UICC	RT	40	48 (5-year OS)	85
	Stage I–IV	NeoCT+RT	40	60	88
Current series	1997 AJCC	NeoCT+RT	90	92.1 (2-year OS)	92.1
	Stage III–IV				

^*^P<0.05.

a For comparison purposes, we estimated a 2-year overall survival rate from the reported survival curve.

RT=radiotherapy, AdjCT=adjuvant chemotherapy, CCRT=concomitant chemoradiotherapy, NeoCT=neoadjuvant chemotherapy, OS=overall survival, RFS=relapse-free survival.

summarises the results. Unfortunately, most studies showed no survival benefit. Because of different staging systems, different prognostic factors, different drugs, and schedules, it is difficult to compare which one is better. Using concurrent chemotherapy of cisplatin 100 mg m^−2^ every 3 weeks during radiotherapy followed by three monthly cycles of PF (cisplatin+5-FU) postradiation adjuvant chemotherapy, the Intergoup study of the United States reported that chemoradiotherapy is superior to radiotherapy alone (Al-Sarraf *et al*, 1998). Of 193 patients with 1987 AJCC/UICC stage III–IV registered, 147 were eligible for analysis. The 3-year progression-free survival (69 *vs* 24%, *P*<0.001) and overall survival (78 *vs* 47%, *P*=0.005) favoured the chemoradiotherapy group. Although this is the only randomised trial of positive results in survival analysis, its wide application to non-American NPC patients should be considered with caution. First, about 30% patients of the Intergroup study have WHO type I histology (keratinising squamous cell carcinoma), but European (Cvitkovic *et al*, 1996), Japanese (Hareyama *et al*, 2002), or Chinese (Chan *et al*, 1995, 2002; Chua *et al*, 1998; Ma *et al*, 2001; Chi *et al*, 2002) series usually contain less than 5% WHO type I patients. Second, the survival data of radiotherapy alone are unexplainedly low–24% 3-year progression-free survival rate and 47% 3-year overall survival rate. The Asian-Oceanian Clinical Oncology Association (AOCOA) trial (Chua *et al*, 1998) presented 42% 3-year relapse-free survival rate and 71% 3-year overall survival for the radiotherapy alone arm. The Hong Kong trial (Chan *et al*, 1995) reported 72% 2-year disease-free survival rate and 80.5% 2-year overall survival rate for patients receiving radiotherapy alone. The Italian trial (Rossi *et al*, 1988) obtained 55.8% 4-year relapse-free survival rate and 67.3% 4-year overall survival rate. A recent nonrandomised study from the Memorial Sloan-Kettering Cancer Center, New York, showed 54% of 3-year progression-free survival and 71% of 3-year overall survival for radiotherapy alone (Wolden *et al*, 2001). A retrospective review from the MD Anderson Cancer Center (Sanguineti *et al*, 1997) covering NPC patients who received radiotherapy alone between 1954 and 1992 also showed a better survival profile than the radiotherapy alone arm of the Intergroup randomised trial.

NeoCT is usually regarded as no benefit at first glance. However, we reconsider it based on a 10-year experience of combined chemoradiotherapy in our hospital and a careful literature review. Our previous study shows that CCRT is better than radiotherapy alone in local control rate and overall survival for patients with 1992 AJCC stage III–IV diseases. Subgroup analysis for very advanced disease (such as selection criteria in this study) revealed no significant difference between CCRT and radiotherapy alone. A phase II study from the Koo Foundation Sun Yat-Sen Cancer Center, Taipei, reported excellent results using CCRT plus adjuvant chemotherapy similar to those of the Intergroup. They reported 96.6% 3-year disease-free survival for 1997 AJCC stage II patients, 87.7% for stage III, but only 51.9% for stage IV (Cheng *et al*, 2000). The editorial comment (Cooper, 2000) recommended that stage IV patients need more effective systemic chemotherapy, such as moving adjuvant therapy to a neoadjuvant position, or adding NeoCT to Cheng's regimen, or inserting another drug with activity in head and neck cancer (e.g. a taxane, mitomycin). A recent study from the National Taiwan University hospital, Taipei, reported 70% 5-year overall survival for stage IV patients using NeoCT of MEPFL (mitomycin, epirubicin, cisplatin, 5-FU, and leucovorin) followed by radiotherapy (Hong *et al*, 2001). The 5-year distant metastasis-free rate of N3a and N3b diseases of AJCC 1997 staging system were 79 and 74%, respectively.

A recent randomised trial from the People's Republic of China allocated 456 patients of the Chinese 1992 staging III/IV disease into 2–3 cycles of PBF (cisplatin, bleomycin, and 5-FU) NeoCT followed by radiotherapy *vs* radiotherapy alone (Ma *et al*, 2001). Although they failed to demonstrate significant benefit in freedom from distant metastasis, there is a trend to favour the chemoradiotherapy group in terms of 5-year relapse-free survival (59 *vs* 49%, *P*=0.05) and overall survival (63 *vs* 56%, *P*=0.11).

There were several nonrandomised studies showing better results of NeoCT. In a matched cohort study from the MD Anderson Cancer Center, significant better 5-year disease-free survival (64±6 *vs* 42±7%, *P*=0.015) and overall survival (69±6 *vs* 48±7%, *P*=0.012) were obtained for the NeoCT group compared with radiotherapy alone (Geara *et al*, 1997). The 5-year cumulative incidence of distant metastasis was 19±5% for the chemoradiotherapy group and 34±6% for the radiotherapy alone group (*P*=0.019). A retrospective study from Korea reported significant better 5-year overall survival (71 *vs* 59%, *P*=0.04) and freedom from distant metastasis rate (84 *vs* 66%, *P*=0.01), favouring NeoCT compared with radiotherapy alone (Hong *et al*, 1999). A larger series containing 618 node-positive NPC patients from the Prince of Wales Hospital, Hong Kong, compared NeoCT plus radiotherapy+adjuvant chemotherapy (*n*=209) with radiotherapy alone (*n*=409). After a median follow-up of 5.5 years, the NeoCT group had significantly less local failures than radiotherapy alone, especially for stage IV patients (Teo *et al*, 1999).

Before changing policy from CCRT with adjuvant chemotherapy to NeoCT for very advanced NPC, we also re-evaluate why some phase III randomised trials could not demonstrate a significant effect of NeoCT. The possible reasons included a relatively lower dose of 5-FU (1000 mg m^−2^ day^−1^ for 3 days infusion instead of 4 or 5 days in other studies) in the Hong Kong trial (Chan *et al*, 1995), a relatively lower dose of cisplatin (120–180 mg m^−2^ person^−1^) in the AOCOA trial (Chua *et al*, 1998), an excess of chemotherapy-related death and of radiotherapy refusal in the International Nasopharynx Cancer Study Group (INCSG) trial (Cvitkovic and INCSG, 1996), or a less advanced stage (Chua *et al*, 1998). In subgroup analysis for patients with bulky neck lymph nodes >6 cm in the AOCOA trial (Chua *et al*, 1998), NeoCT improved 3-year relapse-free survival (63 *vs* 28%, *P*=0.026) and overall survival (73 *vs* 37%, *P*=0.057). The NeoCT arm had a significant better disease-free survival but not overall survival in the INCSG trial (Cvitkovic and INCSG, 1996). If medical care can be improved to avoid excess treatment-related death and good communication can be achieved to reduce radiotherapy refusal, we think that adequate NeoCT followed by radiotherapy may have the potential to improve survival.

Except for the considerations mentioned above, we also think that (1) the timing of NeoCT is better than that of concurrent or adjuvant setting, for example, the best blood supply in the tumour and the best tolerance in the host; and (2) the dose intensity of concurrent chemotherapy during 7–8 weeks of radiotherapy is too low to reduce distant failure. At the beginning of this trial, we initially designed three-phase chemotherapy (neoadjuvant+concurrent+adjuvant) combined with radiotherapy. After the first 17 patients intended to be treated, the compliance to concurrent and/or adjuvant chemotherapy is poor. Thus, we modified our original three-phase chemotherapy to NeoCT plus radiotherapy alone.

Under our unique outpatient weekly F–PL NeoCT, we obtained high CR rate and low toxicity. Clinically and pathologically, CR rates of the primary tumour were 73.3% (66 out of 90) and 62.5% (55 out of 88), respectively. Although the median follow-up (24 months) time is short, a high CR rate has reflected low local recurrence–only 3 of 90 patients with locally advanced tumour (56.7% 1997 AJCC T4, 13.3% T3, and 21.1% T2b disease) suffered from recurrent (2) or persistent (1) primary disease. Other exciting findings of our P–FL NeoCT are rare incidence of mucositis and nearly no hair loss that made patients more comfortable than any other chemotherapy regimen. In all, 47% (42 out of 90) patients had no body weight loss during NeoCT. The outpatient nature of this treatment and lack of alopecia allowed normal social activity and saved more cost. The only weak point we are concerned with is that 10-week NeoCT may be inadequate for eradication of micrometastasis. So far, 16.7% (15 out of 90) distant failure rate is rather good for very advanced NPC. Of 64 (31.3%) NPC patients with the same disease extent who received radiotherapy alone before September 1998 in our department, 20 developed distant metastasis within 2 years of follow-up (unpublished data). Of course, we need a longer time to follow up and evaluate the final outcomes. Now, we consider adding less toxic adjuvant chemotherapy regimen, such as low-dose 5-FU+leucovorin in colorectal cancer. Based on our encouraging results, a phase III randomised trial to compare NeoCT+radiotherapy+adjuvant chemotherapy of low-dose 5-FU+leucovorin with CCRT+adjuvant chemotherapy for very advanced NPC will be initiated in the near future.
